# Asiatic Cholera

**Published:** 1868-04

**Authors:** Philip Harvey

**Affiliations:** Burlington, Iowa


### ASIATIC CHOLERA:

### *A Report on its Nature, Cause, Prevention, and its Cure.*

Reported to the Iowa State Medical Society, at its session in Davenport, May, 1867.

BY PHILIP HARVEY, M. D., OF BURLINGTON, IOWA.

THE subject is one of absorbing interest at the present juncture, from an anticipated invasion of our State by the disease. I do not expect to be able to throw any new light upon it, however. Indeed, when we consider that many of the best minds the world contains have been laboring upon it to very little purpose for half a century, we, in Iowa, possessing no peculiar facilities for its investigation, may well be excused for having but little that is new to offer. Under these circumstances, it is not a subject I should have chosen to treat upon. I shall, however, take it up in the manner prescribed by the Society, and speak firstly of its "Nature," secondly of its "Cause," thirdly of its "Prevention," and fourthly of its "Cure."

*Its Nature.*—By the terms, "Nature of a thing," we mean its essential attributes. Under this head, then, might be included all we have to say upon the subject; but, I shall include under it those remarks only that do not belong to either of the other captions—desiring to conform to the text as given to me. Hindostan may claim the bad eminence of being the "procreant cradle" of this great pestilential scourge of modern times. It seems to have lingered there, endemically, from times quite remote; but, in 1817, it assumed a new phase of activity and mortality, and spread gradually, but irresistibly, into all the surrounding regions. In its course and progress, it has usually followed the principal routes of travel, and is, therefore, supposed to be conveyed by personal or commercial intercommunication. Though retaining an apparent preference for the land of its birth, it has, during the last four decades, become as it were a cosmopolite, and, like the adversary in the Book of Job, has been continually "going to and fro in the earth, and walking up and down in it." In that time it has twice carried its black flag all around the globe, and is now, for the third time, traveling the same extensive circuit. Its march is like that of a devastating conqueror —

> "Amazement in its van and flight combined,
> And sorrows' faded form and solitude behind."

Unrestrained by the power and precautions of governments, it has hitherto disregarded all regulations of quarantine, overstepped lines of circumvallation, and passed through cordons of troops placed around favored localities to stay its progress; it is, therefore, to be presumed its germs may be carried by the air to a considerable though unknown distance. The symptoms of this epidemic scourge are analogous to those of of its sporadic congener—the ordinary cholera morbus—from which its chief differences consist in greater obstinacy, mortality and prevalence. After a premonitory diarrhœa of uncertain duration, usually some twenty-four hours or more, vomiting and purging of a watery fluid, in which floculi of a whitish matter are mixed, sets in. This is usually astonishingly copious, though cases are said to have occurred in which the patients have succumbed with very light discharges, or none at all. These have been called "Cholera Sicca;" but, though I have seen many cases of the disease, I have failed to observe any such. These discharges are accompanied sometimes by severe griping pains, and almost always by severe and painful cramps in the extremities. Though these cramps may come and go with great persistency during the whole course of the disease, and even continue in some degree for near an hour after somatic death. They usually decline in severity as the debility increases. The discharges from the bowels have been sometimes noticed to be tinged with blood and to resemble the washings of meat. After the watery evacuations have continued sometime, usually several hours, though occasionally in much less time, signs of sinking, or collapse, show themselves. The skin then becomes cold, livid and bathed with sweat; the tongue and breath become cold, the features shrunk, the eyes sunken, and the hands soddened like those of women who have been all day at the wash tub. General emaciation takes place, the voice sinks into a hoarse whisper, the pulse becomes more and more feeble and at length quite extinct, and soon thereafter death usually closes the scene. During the progress of these symptoms, the excessive secretion from the skin, stomach and intestines seems to suspend most of the other functions of organic life; the kidneys and liver cease to act, and calorification is arrested. There is usually considerable restlessness and jactitation, and though the skin is as cold as a frog, the patient complains of heat, and throws off the bed clothes. There is incessant thirst, the indulgence of which appetite is quite unavailing, the fluids being thrown up soon after drinking. Considerable muscular power still remains even when the pulse at the wrist is extinct; and though the patient sinks into an apathetic condition, the intellect is mostly retained nearly to the period of death. A remarkable circumstance attending this disease is, that though the body may be quite cold before death, it has been observed to become sensibly warmer for a short time afterwards. This circumstance, and that of the muscular contractions after death, strikingly illustrate the distinction between somatic and molecular death. Many who rally from the collapsed stage of the disease, and have a promise of a happy recovery, yet fall into a febrile and typhoid condition, become comatose and die; though many undoubtedly recover without experiencing this febrile stage, and some by successfully passing through it. These are the most prominent and usual symptoms of this terrible malady as it has occurred within my observation. With regard to *post mortem* appearances it is proper to remark that as this disease is most prone to attack the subjects of previous disease, we find on dissection many pathological indications that have nothing to do with cholera as such. The morbid changes especially due to it I think are chiefly found in the blood, in the mucous membrane of the stomach and intestines, and in local congestions. The blood has been found to be thick, dark colored, and uncoagulable; remaining in both arteries and veins, and being of the same dark and grumous character in both sets of vessels. From this it is evident that it had lost that due crasis necessary to enable it to traverse the capillaries and nourish the tissues. All the viscera, especially the lungs, have been observed to be engorged with it. It seems to have lost its affinities for oxygen and the tissues to be nourished by it, and hence remains dark, cold, and stagnant. Its viscidity is obviously due to the loss of its aqueous parts by excessive secretion, which, with its thinner portion, has also carried off its salts; to this cause also may be due its inability to circulate and to subserve the purposes of vital chemistry. In regard to the morbid appearances presented by the alimentary canal in this disease, the celebrated anatomist, Dr. Horner, of Philadelphia, found the mucous membrane inflamed, softened, or broken down, and in many places covered with a diptheritic deposit, constituting a supposititious membrane. The flakes of lymph observed in the discharges are probably fragments of this deposit. He also found in several cases clusters of vesicles in the stomach and bowels. He says: "In the stomach I found a single bunch resembling a bunch of grapes standing on its base; and in the ileum and colon I found clusters resembling bunches of grapes reposing on their sides. Such clusters had for their nidus, and for connecting them together, a deposit of coagulable lymph." He attributes the disease to a hyperæmia and sloughing of the mucous membrane.

This I consider to be a brief summary of the more important lesions that have been observed in the bodies of those who have died of the disease.

*Its Causation.*—It is supposed to be a zymatic disease; that is, one engendered by its specific cause acting on the blood as a ferment. In this way probably the blood becomes depraved, unsuited to the healthy performance of function, and a source of morbid irritation, resulting in the extravasation of its thinner parts by the emunctories of the alimentary canal. What this specific cause consists in has not been determined; though it seems to be connected with decomposing animal and vegetable matters. The disease does not appear to be contagious in the way that small-pox and measles are; for those who are brought most in contact with it do not take it in a larger proportion than those who keep themselves aloof from the sick. The peculiar germ of the infection, however, does appear to be engendered in the decomposing excretions of those who are suffering from the disease. Stinking sewers and sinks during the period of its prevalence are undoubtedly prolific sources of the disease, as are also the impure waters of streams into which these sewers run. The germ of the disease, in whatever way engendered, may be conveyed to a distance by the air and find a nidus in any collection of decomposing organic matter, especially in sinks and cess-pools, which may then become radiating centres around which the infection will spread its baleful influence.

The predisposing cause of this, as of all other diseases, are countless. Whatever deranges the health may invite an attack. The bodies of those who have died of it are commonly found to bear evidences of other and previous disease. These disturbing influences may be compared to an accumulation of combustible materials; the specific cause, to the spark that ignites them. Great dread of the disease seems to predispose to it, as do excesses of all kinds, and the timid, the depraved and vicious, are most frequently its victims. An addiction to the use of alcoholic liquors, and rich, greasy food, undoubtedly favor its incursion to a much greater extent than the use of fruits and vegetables that are usually more avoided. A deficiency of salt in the food has been looked on as a cause, and I think with reason, for the blood of those who suffer from it is found to be deficient in its normal saline matter. When we consider that chloride of sodium (table salt) furnishes the necessary soda for the formation of bile, which is a saponaceous fluid; and that a stoppage of the biliary secretion is one of the characteristics of the disease, we can easily understand how a deficiency of salt may be connected with an attack of it. It is said that a tax on salt in India diminished its consumption and preceded the great outbreak of the disease in 1817. I have known copious sweating from violent exercise to precede an attack. One of the effects of this secretion would of course be to occasion a loss of the saline matters of the blood. Anything of a septic tendency in the food that is eaten, the fluids that are drank, or the air that is breathed, may prepare the way for the disease. Ozone, which is a natural agent in removing putrefactive matter from the atmosphere, has been found to be deficient in the air during the prevalence of cholera. The subject of Ozone is highly interesting, and deserves, I think, especial notice. There is a mystery about it that has been a source of perplexity to chemists. They talk about ozone and antozone, and are not by any means agreed as to what these substances are. I believe the mystery is occasioned by conferring on "airy nothing a local habitation and a name;" in other words, by giving especial names to what are not especial entities. Ozone I suppose to be nothing more than oxygen in an exalted state of electrical excitation, and antozone the same substance unexcited. The cholera atmosphere is alkaline, and conducive to putrefaction; excited oxygen is an "acid-maker" that neutralizes this alkalinity and counteracts the septic tendency. By the aid of this simple and rational theory the mystery that wraps ozone is solved. Thunder storms are supposed to promote acidification, and to purify the air; the transit of a current of electricity through the air is one of the means of forming what is called ozone. The oxygen and nitrogen of the atmosphere furnish abundance of materials for the formation of acid vapors, and where alkaline gases abound, as in damp and filthy places the production of nitrates or the process of nitrification, as it is called, is observed to take place. Old plaster contains nitrate of lime, and the surface of the ground in many sheltered places is sometimes found covered with an efforescence of nitre: the potash being furnished by the soil, and the nitric acid by the atmosphere under the influence of ozone. The action of this natural antidote appropriately leads us to the consideration of the next head.

*Its Prevention.*—The means of preventing the development or causing the destruction of the cholera germ are entitled to the first consideration under this head. Of these the agents that counteract alkalinity and exert an antiseptic action in those substances which are likely to be productive of the disease are the most effective and reliable. A solution of sulphate of iron thrown into sinks and privies, and sprinkled about the places to be disinfected, is a cheap and valuable preventive, well adapted to general use. A few ounces of coperas dissolved in water, and sprinkled daily by every family about their premises, would doubtless do much to guard against the spread of the disease. A solution of the permanganate of potash of the strength of an ounce to a gallon, or a mixture of carbolic acid and water of one-fourth that strength may be used for like purposes, where it is desirable to avoid leaving the stain of iron rust. The last named antiseptics are among the most efficacious that we possess, but they are not as cheap and abundant as the sulphate of iron. By these latter means infected clothing may be purified. As it is better to prevent than to cure, the means of disinfection should be used wherever needed, whether cholera prevails or not. Drinking water impregnated by organic impurities may be cleansed by an admixture of two or three grains of permanganate of potash to the gallon. In the course of a few hours after the addition of the permanganate the pink hue caused by the salt passes away, and the water is fit for use. The chlorides of lime and soda are not held in such high esteem as disinfectants of the cholera poison as they used to be, and quick-lime, that has been much used for that purpose, is without doubt positively injurious, as possessing an alkaline reagency and promoting the production of ammoniacal vapors and putrefaction in organic substances.

The avoidance of all those things spoken of as predisposing causes of the pestilence is of course to be urged as the *sine qua non* of indemnity from its attacks. The laws of health should be obeyed at all times, but more especially are they demanded when a deadly epidemic rages. I strongly recommend a sparing use of animal and fatty food, with a sufficiency, but not excess, of salt, in cholera times, and am satisfied that a prudent use of wholesome fruits and vegetables is not to be discouraged nor dreaded; on the contrary, they are absolutely necessary to the preservation of health in warm weather. As a rule the higher the temperature of the climate or season, the more should our diet partake of the vegetable character. An observance of this rule is instinctively fallen into by the inhabitants of every region on the globe. Ventillation is an important matter to be attended to in guarding against this disease. The air of close rooms soon becomes alkaline and septic in its tendency, and has frequently been found to favor the spread of cholera. Night air, of which many people have an unreasonable dread, is certainly not so deleterious, if it be so at all, which I doubt, as the air of close and crowded rooms.

*Its Cure.*—It were better to say its treatment—for to treat this disease is, unfortunately, not always to cure it. This head need not detain us long; for, although much has been written, I have very little to say upon it. To discuss the thousand and one methods of cure that have been recommended and failed, would be but waste of time. I shall therefore confine myself merely to that plan of treatment that has been most successful in my hands. I have found the following mixture answer the purpose to check premonitory diarrhœa:

℞.—Tinct. Kino., Syr. Simp. *aa* ℥. *ss.*, Tinct. Opii. ʒ. ij., Chloroform, ʒ. i., Aq. Menth. pip. ℥. *iss.* M. *ft. Mist.*

A teaspoonful to be taken in a little water whenever the diarrhœa is troublesome. Its use might be premised by a mild aperient of castor oil or seidlitz powder, if it should be indicated by a loaded condition of the *primæ viæ;* but for that state of things the looseness itself would be a sufficient remedy in a very short time, under ordinary circumstances. In this early stage of the disease it will generally be sufficient for the patient to lie down, keep moderately warm, observe a very light diet of gruel, or dry toast and tea, and take the astringent and aromatic above recommended. Should the symptoms of veritable cholera have supervened, the use of sugar of lead and opium, in the manner recommended by Dr. Graves, of Dublin, has been successful in putting a stop to the disease in a very large majority of the cases in which I have tried it. Two grains of sugar of lead and a fourth of a grain of opium, given every half hour, or after every discharge from the stomach or bowels, as they become less frequent, I have usually found to put a speedy check to them. I have sometimes combined a grain of calomel and the same of camphor with the dose, but am doubtful if this addition is an improvement. A very large majority of cases in which I have seen this treatment used have progressed favorably, the recovery being rapid. At the same time I have used revellents of mustard, &c., to the abdomen and extremities, and to check vomiting forbidden the use of ingesta of all kinds as much as possible, merely allowing the patient to suck small pieces of ice, or swallow a teaspoonful of water occasionally, to alleviate the tormenting thirst that is commonly experienced. Considering the discharges to be of the nature of a hemorrhage, I should refrain from using stimulants or heating remedies; indeed I am convinced, from what I have seen, that their effect is merely to hasten the case to a fatal termination. There can be no benefit from salines till absorption returns, when there is usually an appetite for something salt; this may be gratified in moderation with propriety. Saline effervescing draughts may be used advantageously to allay the subsequent thirst and fever. Nothing need be said of the special treatment of the subsequent fever or other sequelæ of this disease, as they should be treated, according to the circumstances, on general principles.

